# Factors influencing the attention to home storage of medicines in China

**DOI:** 10.1186/s12889-019-7167-5

**Published:** 2019-06-27

**Authors:** Yin Huang, Lingjie Wang, Changqing Zhong, Shumin Huang

**Affiliations:** 1 School of Logistics and Transportation, Central South University of Forestry and Technology, Changsha, Hunan China; 2 College of Economics and Management, Beijing University of Chemical Technology, Beijing, China; 3 Cardiovascular medicine department, Hunan Provincial People’s Hospital, Changsha, Hunan China; 4 Cardiovascular medicine department, First Affiliated Hospital of Hunan Normal University, Changsha, Hunan China

**Keywords:** Medicine, Home medicine storage, Storage condition, China

## Abstract

**Background:**

Medicines are stored in most households around the world for a range of different purposes including emergency use and the treatment of acute or chronic illnesses. The presence of medicines in households is becoming a significant risk factor for irrational medicine storage, disposal, and use in developing countries due to limited information and knowledge offered on safe storage. This study examined how patients store medicines and highlighted factors which influence home storage behaviours for medicines in China.

**Method:**

A cross-sectional survey of 625 households was undertaken. In each household, data were collected from the head of household present at home. The study covered six provinces in China including the North, Central, and Southern regions. Respondents were interviewed by doctors. The doctors were study assistants and data collectors. “Attention” was taken as the research focus and a five-point Likert scale was used to measure attitudes to medicine storage at home. Factor analysis, variance analysis, and the multivariable logistic regression models were employed in the present study.

**Results:**

Of the households investigated in this study, cold medications were the medicine most commonly stored at home. The majority of the respondents gave more attention to the expiration date of medicines compared to other factors. Only a few respondents thought humidity was important factor influencing home storage of medicines. Despite some attention being given to the location of storage for home medicines, considerably more information is needed to improve awareness. In addition, our data revealed that some attention had been paid to elimination and recycling mechanisms but similarly, increased awareness is needed. There were obvious differences between the populations used in the study, due to differences in socio-demographic characteristics of the study participants. Age is the most important factor influencing the attention paid to home storage of medicines in China.

**Conclusion:**

A major improvement in the awareness of correct storage conditions of medicines for home use can be realized by increased education, and highlighting the importance of correct medicine storage, disposal methods and usages, which has high potential to deliver public health benefits in China. Some suggestions were provided to health care providers.

**Electronic supplementary material:**

The online version of this article (10.1186/s12889-019-7167-5) contains supplementary material, which is available to authorized users.

## Background

In most households around the world, medicines are obtained and stored for various purposes including acute or chronic diseases treatments [1]. These medicines are either prescribed from hospitals or got over-the-counter in the pharmacies [2]. The use of multiple medicines increases many storage problems, such as storing expired medicines at home and under unsuitable storage conditions (i.e. storage environment, location and etc.) [3, 4]. As a direct consequence, patients taking medicines at home may be exposed to adverse effects of improperly stored medicines and treatment failures.

The storage and treatment of medicines should be closely monitored and strictly regulated at all the stages of the medical supply chain according to the Good Distribution Practice Guidelines [5, 6]. However, in most developing countries, limited knowledge exists amongst populations on the safety of medicines storage commonly found in homes [1, 7, 8], often leading to irrational medicine use. Only a few studies have investigated different aspects of adequate storage conditions and factors affecting medicine home storage practices. By specifically investigating home storage practices and its impacts on patients, this study aimed to provide more insight into home storage practices, taking into account a combination of important storage requirements including environments, locations and disposal methods.

In the past 20 years, China has experienced unprecedented socio-economic development, but its level of medical supervision lags behind that of many western countries. This is mainly due to its inadequate health infrastructure including insufficient medical supplies, doctors and pharmacists compared with China’s large population. It is difficult for the medical product suppliers and doctors to supervise all uses of medicines in the home. The practice of storage of medicines in homes in such environments is more likely to be seen as an avenue to improve access to medicines and immediate health care among the population: however, the implications of having medicines in homes have not been fully quantified in most parts of the world, especially in China. We found few studies on home storage of medicines in China. This study therefore explored factors that predict the storage of medicines in households in China.

## Methods

### Study design

A cross-sectional household survey was carried out between March and April 2018 in China. The study covered six Chinese provinces and municipalities including: Beijing (the capital of China), Hebei Province (located in North China), Hubei and Hunan Provinces (located in central China), Shanghai (located in East China) and Guangdong Province (located in South China). Respondents were interviewed by doctors. The doctors were study assistants and data collectors. The respondents gave their written informed consent about the study and were asked to respond by giving written answers.

### Questionnaires and data collection

For questionnaire design, Q8 (expiry date) and Q9 (manufacture date) are posed on the basis of work by Ocan et al. [1], Q10 (storage temperature), Q11 (cold storage), Q12 (storage humidity), and Q13 (preservation of medicine drying) are posed on the basis of work by Velieland et al. [5], Q14 (keeping medicines away from the children), Q15(keeping medicines out of kitchen and bathroom), Q18 (eliminating medicines which had expired) and Q19 (special recycling mechanisms) are posed on the basis of work by Ocan et al. [1] and Velieland et al. [5]. Q16 (placement of first aid medicines) and Q17 (medicine mixing) (the detailed questionnaire please see Additional file 1) are suggested by collating opinions among 20 households (20/65) in the pre-tested households (65/625) and 20 specialists.

Data were collected using a structured questionnaire. The questionnaire was pre-tested on sixty-five households (65/625) in Hunan province and data were collected from the head of each household (only one person), when present at home. Moreover, the questionnaire was sent to twenty specialists including doctors from the top three hospitals, including Hunan Provincial People’s Hospital and Xiangya Hospital of Central South University, pharmacists, Professors in medical schools, including the Medical School of Hunan Normal University and Xuzhou Medical College, and medical logistics specialists from Central South University of Forestry and Technology as well as Beijing University of Chemical Technology. They were independent of the present research. Useful information that had not been captured by the original questionnaire was incorporated by developing additional questions, such as Q16 and Q17. The reliability of questionnaire was improved by the pre-tested households (65/625) and 20 specialists.

In contrast to other previous studies which assume the storage status of home medicines as a measurement standard, our study considered a new viewpoint and used a 5-point Likert type scale to measure the attitudes to home medicine storage. The measurement items were quantified on a 5-point Likert type scale with 5 being rated as attracting the most attention and 1 being rated as attracted no attention. “Attention” which was used in our study means the level to which survey respondents pay attention to the home storage of medicines. It was used to evaluate the respondents’ medicine home storage behaviors. A total of 20 questions were used in the survey to determine the factors influencing the attitudes to home medicine storage.

A survey was conducted in the communities of six provinces in China by sending the questionnaires to different Chinese households. In each household, data were collected from the head of each household (only one person) when present at home between March and April 2018. We have obtained written informed consent from all respondents before undertaking the survey.

### Data analysis

SPSS 21.0 was used for data analysis. The descriptive statistics was used to research the demographic characteristics of the respondents and then factor analysis was conducted. Reliability analysis was checked by calculating the Cronbach’s alpha value. The validity of the questionnaire was measured using the Kaiser-Meyer-Olkin (KMO) and Bartlett’s Test of Sphericity.

The validity of this study was ensured by the accurate construction of the questionnaire which was then evaluated by 20 specialists. These specialists were selected according to three rules: first, they were selected from famous hospitals and medical schools in China, second, they were familiar with the nature of the home storage of medicines in China, and third, they were professors with excellent reputations in the disciplines of medicine and pharmacy. The Cronbach’s alpha-value for all the factors was determined to be 0.848, where a Cronbach’s alpha-value above 0.70 indicates that the output is reliable [9, 10]. The KMO referred to the suitability of the sampling for the present analysis, where the KMO value was equal to 0.865, which was larger than the suggested minimum values of 0.6 [9, 10]. The KMO value was reported as being high (0.865) which requires subsequent factor analysis. The results of principal component analysis show that among all factors, the characteristic values of factors 1 to 5 vary greatly, and the preliminary judgment of the composition of the five factors can explain the accumulated variance of 72.154%, which was larger than the suggested minimum value of 70% [9, 10]. The factor structures for the items used to construct the scale supported the conclusion that the constructs described specific medicine home storage behaviors because the values of rotated components were greater than 0.6, which was larger than the suggested minimum value of 0.5 [9, 10]. The reliability of the scale was thus deemed acceptable.

## Results

### Sample profile

With the assistance of community service managers, we randomly sampled 1000 households from the communities according to community population information registration forms. At the end of the period, after removal of incomplete responses, a total of 625 completed and usable questionnaires were received, equating to a response rate of 62.5% (625/1000). The following assumptions and calculations were considered according to simple random samples. Our final usable questionnaire size (625) is larger than the calculated sample size (594) and the confidence level is 95%. Therefore, the sample size is sufficient to reflect the overall effect of the sample (the detailed study data please see Additional file 2).

The majority of respondents, 61.9% (387/625) in the households visited were females. A high proportion 60.6% (379/625) of the respondents were employees from different companies. In terms of age groups, 78.4% (490/625) of respondents were less than 30 years old, and 12.0% (75/625) of the respondents were aged between 31 and 45. A total of 441 respondents reported having a university degree (70.6%), followed by master’s degree or above (1.8%, 11/625), senior high school (7.8%, 49/625), junior high school (6.6%, 41/625) and education level below junior high school (13.3%, 83/625). In terms of family members, 34.2% (214/625) of households had four members, and 31.0% (194/625) of households had five or above members (Table 1).

**Table 1:** Socio-demographic characteristics of respondents

Characteristic	Number of respondents (*n*=625)		
	Description	Number (*n*)	Proportion (%)
Sex	Female	387	61.9%
	Male	238	38.1%
Age	18-30 years	490	78.4%
	31-45 years	75	12.0%
	46-60 years	39	6.2%
	61-75 years	17	2.7%
	Above 75 years	4	0.6%
Education level	Below junior high school	83	13.3%
	Junior high school	41	6.6%
	Senior high school	49	7.8%
	University degree	441	70.6%
	Master’s degree or above	11	1.8%
Occupation	Housewife	23	3.7%
	Teacher	27	4.3%
	Farmer	43	6.9%
	Civil servant	12	1.9%
	Company employee	379	60.6%
	Worker	28	4.5%
	Engineer	45	7.2%
	Self-employed	24	3.8%
	Others	44	7.0%
Number of members in household	1	9	1.4%
	2	22	3.5%
	3	186	29.8%
	4	214	34.2%
	5 or above	194	31.0%

### Prevalence of home storage of medicines

Medicines kept in households were mainly sourced from drugstores (60.0%, 375/625). Other medicines (36.8%, 230/625) were obtained from hospitals and 3.2% (20/625) were gifts from friends and relatives. The medicines investigated in the survey were classified according to the Chinese Pharmacopoeia (2015): we chose 14 types by investigation of several large hospitals and pharmacies. Some medicines that are not often used or purchased have been removed following suggestions from the 20 specialists who evaluated the questionnaire. Cold medication (86.1%) was the most common category of medicines kept in households. Specifically, the following were the major classes of medicines found in the households: gastrointestinal medicines (27.0%), pain medications (22.9%), vitamins (20.6%), antibiotics (19.0%), external painkillers (16.5%) and external anti-inflammatory antidotes^1^ (15.4%) (Table 2).

**Table 2:** Major classes of medicines found in Chinese households

Major classes	Storage		No storage	
	Number (*n*)	Proportion (%)	Number (*n*)	Proportion (%)
Cold medication	538	86.1%	87	13.9%
Pain medication	143	22.9%	482	77.1%
Antibiotics	119	19.0%	506	81.0%
Sleep aids	27	4.3%	598	95.7%
Antiallergic medicines	61	9.8%	564	90.2%
Gastrointestinal medicines	169	27.0%	456	73.0%
Digestive medicine	104	16.6%	521	83.4%
External painkillers	103	16.5%	522	83.5%
External anti-inflammatory antidotes	96	15.4%	529	84.6%
Medicine for the treatment of gynaecological conditions	30	4.8%	595	95.2%
Vitamin supplements	129	20.6%	496	79.4%
Medicines for hypertension and coronary heart disease	47	7.5%	578	92.5%
Quick-acting heart-saving pills, such as nitroglycerin	31	5.0%	594	95.0%
Others	87	13.9%	538	86.1%

### Storage and disposal of medicines kept in households

The level of attention to the home storage of medicines in the households is shown in Table 3.

**Table 3:** The attention to the home storage of medicines kept in the households

Question	Number of respondents (*n* = 625)		
	Attention levels	Number (*n*)	Proportion (%)
Q8	Most attention	234	37.4%
	More attention	178	28.5%
	Attention	133	21.3%
	Some attention	53	8.5%
	No attention	27	4.3%
Q9	Most attention	236	37.8%
	More attention	180	28.8%
	Attention	122	19.5%
	Some attention	54	8.6%
	No attention	33	5.3%
Q10	Most attention	49	7.8%
	More attention	100	16.0%
	Attention	166	26.6%
	Some attention	136	21.8%
	No attention	174	27.8%
Q11	Most attention	120	19.2%
	More attention	150	24.0%
	Attention	158	25.3%
	Some attention	93	14.9%
	No attention	104	16.6%
Q12	Most attention	49	7.8%
	More attention	63	10.1%
	Attention	162	25.9%
	Some attention	126	20.2%
	No attention	225	36.0%
Q13	Most attention	95	15.2%
	More attention	133	21.3%
	Attention	185	29.6%
	Some attention	99	15.8%
	No attention	113	18.1%
Q14	Most attention	192	30.7%
	More attention	167	26.7%
	Attention	157	25.1%
	Some attention	54	8.6%
	No attention	55	8.8%
Q15	Most attention	266	42.6%
	More attention	178	28.5%
	Attention	107	17.1%
	Some attention	40	6.4%
	No attention	34	5.4%
Q16	Most attention	120	19.2%
	More attention	135	21.6%
	Attention	177	28.3%
	Some attention	90	14.4%
	No attention	103	16.5%
Q17	Most attention	97	15.5%
	More attention	84	13.4%
	Attention	162	25.9%
	Some attention	118	18.9%
	No attention	164	26.2%
Q18	Most attention	77	12.3%
	More attention	148	23.7%
	Attention	228	36.5%
	Some attention	146	23.4%
	No attention	26	4.2%
Q19	Most attention	165	26.4%
	More attention	120	19.2%
	Attention	163	26.1%
	Some attention	85	13.6%
	No attention	92	14.7%

The expiration date of medications is a vital factor in the home storage, including expiration and manufacturing dates [1, 2, 5] (from Q8 to Q9). The results of Q8 and Q9 showed that the respondents paid great attention to the expiration date of medicines. The majority of the respondents gave much attention to the expiration date of medicines.

The storage environment was one of the most important factors influencing medicine home storage [1, 2, 5] (from Q10 to Q13). For storage temperature (Q10), only 7.8% of the households (49/625) gave most attention, whilst 27.8% (174/625) paid no attention to storage temperature. For refrigerated medicines (Q11), 19.2% (120/625) of respondents gave most attention to cold storage and 24.0% (150/625) gave more attention to it, whilst 16.6% (104/625) gave it no attention. The results of Q10 and Q11 showed that the respondents paid some attention to the medicine storage temperature, but not enough. Storage humidity was a factor that could be easily ignored (Q12). Only 7.8% (49/625) of the households thought that storage humidity needed most attention, whilst 36.0% (225/625) of the households thought it needed no attention. Another factor which was easily neglected was dry preservation of medicine (Q13). Many medicines will lose their potency if allowed to absorb moisture: because this causes hydrolysis thus rendering them ineffective. 15.2% of households (95/625) thought that dry preservation of medicine needed most attention, whilst 18.1% of the households (113/625) thought it needed no attention. From the results of Q12 and Q13, only a few respondents thought humidity in the environment was important for the home storage of medicines. Some respondents did not even know what the dry preservation of medicine was and how it was impacted during medicine storage.

Location played an important role in the home storage of medicines [1, 2] (from Q14 to Q17). The majority of the households (30.7%, 192/625) gave most attention to keeping medicines away from the children (Q14) with only 8.8% of respondents (55/625) paying no attention to it. 42.6% of households (266/625) thought that medicines kept out of kitchen and bathroom (Q15) needed most attention, and 28.5% of households (178/625) thought it needed more attention. Only 5.4% (34/625) paid no attention to it. However, only 19.2% (120/625) paid most attention to the placement of first aid medicines (Q16) and 21.6% (135/625) paid more attention to it, whilst 16.5% (103/625) paid no attention to it; however, it is vital to medicine home storage because in the daily life we often encounter a variety of patients with acute illnesses. If we cannot give the medicines to the patients in the first place, the consequences would be unimaginable. 26.2% of households (164/625) had never paid attention to mixing of medicines (Q17), for example, internal and external medicines or adult and child medicines, whilst only 15.5% (97/625) paid most attention to these problems. For external medicine, although it is safe to use it topically, it is often irritating, corrosive, or toxic if taken orally. For children’s medicine, the doses given to children vary greatly from those recommended for adults. If put together with adult medicine, the child may take the wrong medicine or be overdosed. From the results to Q14, Q15, Q16 and Q17, some attention had been given to the location of home medicine storage, which was still not sufficiently adequate.

Procedures to deal with medicine which had expired or whose traits had changed significantly are an important factor in medicine storage and disposal (Q18 and Q19). However, only 12.3% (77/625) of households thought that eliminating medicines which had expired (Q18) needed most attention, while 23.4% (146/625) of households thought it needed some attention, and 4.2% (26/625) of households considered it needing no attention. When a special recycling mechanism (Q19) was required for medicine storage and disposal, 26.4% (165/625) of the households paid most attention and 19.2% (120/625) of the households paid more attention to it. However, special recycling mechanisms attracted no attention from 14.7% (92/625) of households. Expired medicines are likely to lead to medicine abuse and environmental pollution. From the results of Q18 and Q19, some attention had been paid to medicines which had expired, but still needed more attention.

### The differences of medicines home storage

Variance analysis and comparison of mean values were employed to reveal the differences of home storage of medicine. Cross-over analysis was used to explore the causes of any observed differences (Table 4).

**Table 4:** Results of variance analysis and comparison of mean values

	Gender	Educational background	Age	Occupation	Family members
Q8	0.108	0.110	0.123	0.311	0.690
Q9	0.967	0.217	0.147	0.166	0.769
Q10	0.019*	0.132	0.009**	0.022*	0.046*
Q11	0.056	0.097	0.000***	0.001**	0.135
Q12	0.000***	0.000***	0.000***	0.119	0.673
Q13	0.001**	0.026*	0.004**	0.113	0.548
Q14	0.302	0.006**	0.000***	0.000***	0.527
Q15	0.845	0.289	0.020*	0.259	0.609
Q16	0.005**	0.102	0.033*	0.191	0.678
Q17	0.000***	0.007**	0.022*	0.216	0.129
Q18	0.199	0.001**	0.006**	0.028*	0.218
Q19	0.547	0.000***	0.003**	0.004**	0.367

***p < 0.001; **p < 0.01; *p < 0.05

### 3.4.1 Gender

As shown in Table 4, significant differences were found between different genders in the attention paid to storage temperature (Q10), storage humidity (Q12), medicine moisture-proofing (Q13), placement of first-aid medicines (Q16), and mixed medicine storage problems (Q17). According to comparison of the mean values between different genders, it was found that men paid more attention to the storage safety of medicine compared to women which attributed to difference of their educational backgrounds. The percentage of male respondents with university degrees or above was slightly higher than that of female respondents (73.9% compared with 71.3%). The percentage of male respondents with junior high school level of education or below was slightly lower than that of female respondents (17.6% compared with 21.2%). In China, men have easier access to better education, and so the educational background of male respondents was better than that of females.

### Educational background

The respondents who had been educated to university level of education or above, may have more information and knowledge about medicine safety and also be more eager to learn. Through the analysis of variance, significant differences were found amongst different educational levels concerning attention to storage humidity(Q12), moisture-proofing(Q13), safe positioning away from children(Q14), mixed medicines placement problems(Q17), medicine elimination (Q18) and recycling mechanisms (Q19) (Table 4). According to the mean values, the comparison amongst different educational levels shows that the respondents who had a master’s degree or higher, paid more attention than the others. These data indicated that educational background may affect home medicine storage practices.

### Age

Through the analysis of variance, significant differences were found among different age groups in their attention to storage temperature(Q10), cold storage(Q11), storage humidity(Q12), moisture-proofing(Q13), safe positioning away from children(Q14), placement out of the kitchen and bathroom (Q15), first-aid medicine placement (Q16), mixed medicine placement problems(Q17), medicine elimination (Q18) and recycling mechanisms (Q19) (Table 4). Amongst the different age groups, we found that the respondents aged between 61 to 75 years gave most attention to medicine storage safety (the mean values are highest in most responses). These data may be related to the fact that elderly respondents used more multiples of medicines and were more likely to acquire information and knowledge on the safety and management of medicines at home. Also, it is widely reported that medicine use increases with age, and it was estimated that 25–40% of people aged above 65 use at least five medicines [5]. We found that most of the attention on home medicine storage increased from 18 to 75 years old, but after 75 years of age, attention declined with age (Additional file 1).

Information and knowledge concerning the home storage of medicines increased with age. The increased information and knowledge on home storage practices could help the respondents identify which medicines and aspects of home storage need more attention.

### Occupation

From our data, significant differences amongst different occupations in the attention of storage temperature (Q10), cold storage (Q11), safe positioning away from children (Q14), medicine elimination (Q18) and recycling mechanisms (Q19) were observed by analysis of variance (Table 4). For storage temperatures, medicine elimination and recycling mechanisms, the mean values of civil servants were highest. For safe positioning away from children, the mean value of housewives was highest. From the results, we found that all the civil servants had good educational backgrounds: 75.0% of them had university level of education or above. The categories of medicines possessed by housewives are most diverse among all occupations, accounting for 85.7% (12/14) of all categories of medicines that are stored at home in China.

### Family members

As shown in Table 4, significant differences were found between different family members in the attention paid to storage temperature (Q10). The results showed that the number of family members was associated with medicine home storage temperature behaviors.

### Multivariable logistic regression of the factors influencing the attention to home storage of medicines in China

The multivariable logistics regression models showed that age is the most important factor influencing the attention to home storage of medicines in China (Q8, Q10, Q11, Q12, Q13, Q 14, Q15, Q17 and Q19 are positively affected by age). Gender is shown to be the second most important factor affecting medicine storage behavior (Q10, Q12, Q13, Q16 and Q17 are positively affected by gender). And occupation is the third most important factor affecting medicine storage behavior (Q9 and Q18 are positively affected by occupation) (Results of multivariable logistic regression please see Additional file 3).

## Discussion

### Attention on the prevalence of home storage of medicines

The information from this study is of importance to healthcare professionals and the community due to risks associated with the presence of medicines in homes. The inappropriate use of medicines may lead to emergence of drug resistance where antimicrobials are misused, or the more general emergence of adverse drug reactions. In the present survey, we found that cold medication (86.1%) was the most common category of medicines kept in households. According to Okumura et al. [2], antibacterial drugs (40.1%) are the most common medicines used in old Dutch patients, however, the difference in disease spectrum and epidemiological features in different countries means that multiple types of medicines are being used in diverse fields and with different probabilities of prescription. The Chinese residents were likely to store more medicines at home than required, especially for some common categories such as cold medication (538/625). As a result, more home storage problems arise such as drug resistance and adverse drug reactions. This is a universal problem in some countries [11–13]. These studies showed that storing a large quantity of medicines at home exposed patients to adverse drug effects and treatment failure.

The high prevalence of cold medications in households could indicate widespread use of these medicines in Chinese communities, which is consistent with the findings of some other previous studies [14, 15] which showed cold medications were the most common category of medicines kept in households. Cold medication is a universal OTC medicine in China used to relieve cold symptoms. To relieve cold symptoms, large quantities of medicines are stored and some of them may be no longer used. This may result in poorer medicine home storage behaviors, such as taking time-expired medicines. Medicine abuse may be a global problem, especially in some developing countries such as Vietnam [2]. In our study, the most serious problem in medicine abuse is self-medicating without professional medical guidance and taking medicine beyond the scope of medical care and dose standards. Cold medication abuse is one such practice, and may be one of the most serious medicine abuse problems in China.

### Attention to expiration date

The expiration date on medicines indicates the end of the useful life of the medication. The mean duration of storage was approximately ten weeks which ranged from 0 to 93 weeks [16]. The respondents paid great attention to expired medicines but there was also little control over the expired medicines in China which is a persistent problem. Expired medicines may cause adverse reactions: for example, expired tetracycline may cause toxicity and affect liver and kidney function. Taking them after expiration may cause liver and kidney damage. Strategies for reducing unused or expired medicines are required to specifically target health professionals such as physicians and pharmacists [8]. Health professionals need to minimize prescribing practices by reducing excessive quantities of medicines and/or the use of unnecessary medicines to promote healthier lifestyles [11].

### Attention to the storage environment

With limited knowledge of the proper storage conditions and practices, appropriate storage for medicines in the communities, the presence of medicines in households is likely to fuel irrational medicine use resulting from unintentional use among household members. For example, insufficient attention was paid to storage temperature in the survey. For the storage of refrigerated medicines, temperature between 2 and 8 °C is necessary to prevent medicine from rapid degrades [5]. Refrigerators should be equipped with temperature registration and controls, also the use of dedicated refrigerators for medicines storage should be encouraged: however, only a few respondents paid attention to. A lack of related knowledge may be the main reason for this. Adverse medicine reactions may occur due to improper storage temperature. For example, antibiotics such as penicillin cephalosporin have poor stability and are easily weakened or rendered ineffective under improper storage temperature conditions. Taking them may cause the delay of disease progression: this finding is comparable with other reports [1, 17] which considered storage temperature to play an important role in medicine home storage. They showed that medicines not requiring refrigeration were considered to require storage at room temperature defined as a temperature below 25 °C and refrigerator storage was considered adequate if the temperature was between 2 and 8 °C.

From our survey, humidity was a factor that was easily ignored. Only a few respondents thought storage humidity and dry preservation were important for the home storage of medicines. However, improper storage without effective dry preservation could accelerate the degradation of medicines and advance expiration. For example, some medicines are deliquescent: some are hygroscopic, such as potassium chloride and acarbose. Some medicines are deliquescent because of their form, such as sugar-coated, or effervescent, tablets, however, health professionals and pharmacies are focused on disseminating information to patients on the use of medicine with limited information offered on safe storage [7], in particular concerning storage humidity and dry preservation. The improper storage of medicines in humid environments was inherently associated with medicine degradation and some adverse drug reactions. These findings are consistent with the study previously reported [18] which considered medicines should not be stored in a humid place to prevent physico-chemical changes thereto. Here, we defined these changes as deliquescence reactions.

### Attention to home storage location

The location of home storage of medicines is also an important factor worthy of investigation. Storing multiple medicines in wrong locations is poor medication storage practice [3, 19]. The unorganized storage of medicines in improper places in households may lead to accelerated degradation, health hazards to children and wasted resources [12]. The majority of the households paid the most and more attention to keeping medicines away from children as well as out of kitchens and bathrooms. Most households had a medicine cabinet to keep medicines out of the reach of children. A high proportion of households considered the kitchen and bathroom as unadvisable sites for keeping medicines as they were at risk of exposure to high temperature and humidity. Again, these findings are in agreement with those from another previous study [12]. Sharif et al. gave examples to verify the importance of medicine home storage location. They pointed out that keeping medicines in a drug cabinet away from the reach of children is highly recommended, and that kitchens and bathrooms are unadvisable places in which to store medications as they are at risk of exposure to high temperature and humidity that accelerate the onset of their instability, however, few respondents paid attention to medicine home storage location in their study [12]. In our study, the respondents paid more attention to these locations. This may show that Chinese people may be more concerned with the medicine home storage location compared to those elsewhere.

First aid medicine placement and medicine mixing problems have not received enough attention in our survey. First aid medicine placement is very important for emergency use but only attracted little attentions. It is a dangerous factor for some patients with serious diseases such as cardiovascular conditions. 7.5% (47/625) and 5% (31/625) of the respondents stored hypertension/coronary heart disease medicines and quick-acting heart-saving pills, such as nitroglycerin, but only 21.3% (10/47) and 19.2% (9/47) of the respondents paid most and more attention to first aid medicine placement. Only 32.3% (10/31) and 22.6% (7/31) of respondents paid most and more attention to it. It showed that if a heart attack occurs, the patient is unable to find emergency medicine, such as nitroglycerin, their life will be threatened.

It has been reported that causes of death in some diseases, such as cardiovascular diseases, have complex mechanisms and may be impacted by social factors [20] such as medicine home storage behaviors. Many elderly patients with cardiovascular diseases used multiple medicines and have more difficulties with medicine management at home because of limited knowledge for proper home storage practices [3, 21], however, we have not found any studies related to first aid medicine placement and medicine mixing problems. We call on the medical profession to pay attention to first aid medicine placement and medicine mixing problems to reduce mortality from cardiovascular and other diseases.

### Attitudes to medicine disposal

Attitudes towards the disposal of medicines play a vital role in home storage, in particular for expired medicines. Expired medications in the home pose a serious health and safety risk. However, there is no special recycling mechanism in China. About 80% of respondents have never received any information about appropriate medicine disposal and few paid attention to special recycling mechanisms. These findings are in accordance with those from a previous study [4, 21], which suggest that special medicine recycling mechanisms are urgently required in China. Lacking an effective recycling mechanism will lead to illegal medicine acquisition and environmental pollution. Greater access to convenient disposal sites such as community pharmacies would help control inappropriate medicine disposal.

### Differences in socio-demographic characteristics

Socio-demographic characteristics play an important role in the home storage of medicines [2, 22]. The previous study [2] showed that there was a difference between sample populations in terms of socio-demographic characteristics. On the basis of the multivariable logistic regression models, gender was the second most important factor affecting medicine storage behavior. And from the variance analysis and comparison of mean values of the socio-demographic characteristics, we found that men paid more attention to the storage safety of medicines rather than women because of educational background difference which was higher for men, particularly in rural areas of China. The effect of education level on home medicine storage was also proven in our study. Sorensen showed that female gender was associated with some poorer practices in medication management at homes, but did not give the reason [3]. Our study showed that female gender was associated with poorer medicine home storage behavior because of the associated poorer educational background, therefore, knowledge and educational level were considered to be a factor that could not be neglected [2, 22].

In contrast to the previous study [2], in terms of educational status, there was obvious difference between the sample populations. In our study, the respondents who had master degrees or above were found to pay more attention than others by variance analysis and mean values comparison. Good medicine home storage, disposal and usage behaviors belong to the scope of health awareness. Better educational background of the patients would lead to more rational medicine-use with higher awareness [17]. Health awareness increases with educational level and a high level of education may increase the need to maintain one’s health. These findings are in accordance with the findings of previous studies [17, 22] which considered educational background as associated with medicine storage behaviors.

Age is the most important factor in medicine home storage behavior according to variance analysis and multivariable logistic regression models. We found that the attention to home storage of medicines increases with ages because of the increase of diseases and medicine usage. There may be an association between duration of use, and storage knowledge and practices [23], which is in accordance with a previous report [5], which considered older patients were more compliant with the storage criteria; however, in our study attention declined after 75 years of age (Attention to home storage of medicines amongst different age groups please see Additional file 4). It may be due to their decreased visual and cognitive impairment.

Occupation is also an important factor that is easy to be ignored in home storage behavior of medicines and few studies have focused on this aspect [2]. Okumura indicated that mothers play an important role in home storage practices [2], which was confirmed in our study. The housewife respondents paid most attention to the several medicine home storage behaviors including cold storage and placing medicine in a location away from children. This may be due to the central role that women, as housewives, play in maintaining the health of family members, a practice which is common in some parts of the world [2] which considered the attitude and knowledge of housewives would modify home use and storage behaviors at household level.

## Conclusions

With the gradual increase in the demand for medicines and increasingly extensive self-diagnosis and self-medication, the variety and quantity of medicines stored in households is increasing. The storage and disposal of household medicines have been becoming an important issue in health public awareness; however, patients may be obtaining some medicines in pharmacies or communities without appropriate information and knowledge related to correct and safe storage. Therefore, there is a great need to educate and motivate people to have correct medicine storage, disposal and usage towards improving public health in China. Health care providers have a captive audience at the point of prescribing and may be able to educate patients effectively. Extra, and repeated, information, both written and oral, on medicine storage conditions (e.g., paying attention to first aid medicine placement and medicine mixing problems) should be provided to patients as deemed necessary for specific medicines.

## Additional files

Additional file1: Questionnaire. The questionnaire which was developed for this study. (DOCX 19 kb)

Additional file 2: Study Data. All the data used in our study. (XLS 170 kb)

Additional file 3: Results of multivariable logistic regression. Multivariable logistic regressions of the factors influencing the attention to home storage of medicines in China. (DOCX 17 kb)

Additional file 4: Attention to home storage of medicines amongst different age groups. Table listing attention to home storage of medicines amongst different age groups. (DOCX 22 kb)

## Data Availability

All the data used in our study have been contained within the additional files.

## Abbreviations

From Q1 to Q19: from Question 1 to Question 19
KMO: Kaiser-Meyer-Olkin
OTC: Over the counter
Sig value: Significance value

## Footnotes

1 External painkillers are mainly used for bruising injury, blood stasis, rheumatism, and joint pain. Most of them are sprays, such as Yun nan bai yao spray. And external anti-inflammatory antidotes are mainly used for swollen furunculosis, such as acne ointments.
