# Supplementary material for: Factors influencing the attention to home storage of medicines in China
Source: BMC Public Health. 2019 Jun 27;19:833. doi: 10.1186/s12889-019-7167-5 (PMC6598263; doi:10.1186/s12889-019-7167-5)
Supplement: Supplementary file 1 — Questionnaire. The questionnaire which was developed for this study. (DOCX 19 kb) [file 12889_2019_7167_MOESM1_ESM.docx]

**Questionnaire of English version**

**Personal information**

Q1. **Sex**: A. Male B. Female

Q2. **Age:** A. Less than 30 years old B. Between 31 and 45 years old

C. Between 46 and 60 years old D. Between 61 and 75 years old

E. 75 years old or older

Q3. **Educational background**: A. Below junior high school B. Junior high school

C. Senior high school D. University degree E. Master’s degree or above

Q4. **Occupation**: A. Housewife B. Teacher C. Farmer D. Civil servant

E. Company employee F. Worker G. Engineer H. Self-employed

I. Other

Q5. **Number of family members:** A. Only one member B. Two members

C. Three members D. Four members E Five members or above

Q6. **City**: A. Beijing B. Hebei C. Hubei D. Hunan E. Shanghai F. Guangdong

Q7. **Home storage of medicine types: (Multiple Choice)**

A. Cold medication

B. Pain medication

C. Antibiotics medicine

D. Sleep aids

E. Antiallergic

F. Gastrointestinal medicines

G. Digestive medicine

H. External painkillers

I. External anti-inflammatory antidotes

J. Medicine for the treatment of gynecological conditions

K. Vitamin

L. Medicines for hypertension and coronary heart disease

M. Quick-acting heart-saving pills, such as nitroglycerin

N. Others

Q8. **To what degree did you pay your attention on the expiry date of medicines?**

1. No attention B. Somewhat attention C. Attention D. More attention

E. Most attention

Q9. **To what degree did you pay your attention on the manufacture date** **of medicines?**

1. No attention B. Somewhat attention C. Attention D. More attention

E. Most attention

Q10. **To what degree did you pay your attention on the storage temperature** **of medicines?**

A. No attention B. Somewhat attention C. Attention D. More attention

E. Most attention

Q11. **To what degree did you pay your attention on the cold storage** **of refrigerated medicines?**

A. No attention B. Somewhat attention C. Attention D. More attention

E. Most attention

Q12. **To what degree did you pay your attention on the storage humidity of medicines?**

A. No attention B. Somewhat attention C. Attention D. More attention

E. Most attention

Q13. **To what degree did you pay your attention on the preservation of medicine drying?**

A. No attention B. Somewhat attention C. Attention D. More attention

E. Most attention

Q14. **To what degree did you pay your attention on keeping medicines away from the children?**

A. No attention B. Somewhat attention C. Attention D. More attention

E. Most attention

Q15. **To what degree did you pay your attention on keeping medicines out of kitchen and bathroom?**

A. No attention B. Somewhat attention C. Attention D. More attention

E. Most attention

Q16. **To what degree did you pay your attention on the placement of first aid medicines?**

A. No attention B. Somewhat attention C. Attention D. More attention

E. Most attention

Q17. **To what degree did you pay your attention on the medicine mixing?**

A. No attention B. Somewhat attention C. Attention D. More attention

E. Most attention

Q18. **To what degree did you pay your attention on eliminating medicines which had expired?**

A. No attention B. Somewhat attention C. Attention D. More attention

E. Most attention

Q19. **To what degree did you pay your attention on the special recycling mechanisms for medicine disposal?**

A. No attention B. Somewhat attention C. Attention D. More attention

E. Most attention

Q20. **The** **medicines kept in households were mainly sourced from ( ).**

A. drugstores B. hospitals C. friends and relatives
