# Supplementary material for: Factors influencing the attention to home storage of medicines in China
Source: BMC Public Health. 2019 Jun 27;19:833. doi: 10.1186/s12889-019-7167-5 (PMC6598263; doi:10.1186/s12889-019-7167-5)
Supplement: Supplementary file 3 — Results of multivariable logistic regression. Multivariable logistic regressions of the factors influencing the attention to home storage of medicines in China. (DOCX 17 kb) [file 12889_2019_7167_MOESM3_ESM.docx]

**Additional file 2 Results of multivariable logistic regression**

|  | **Q8** | **Q9** | **Q10** | **Q11** | **Q12** | **Q13** | **Q14** | **Q15** | **Q16** | **Q17** | **Q18** | **Q19** |
| --- | --- | --- | --- | --- | --- | --- | --- | --- | --- | --- | --- | --- |
| **Sex** | 0.102 | 0.803 | 0.024* | 0.059 | 0.000*** | 0.001** | 0.270 | 0.968 | 0.008** | 0.000*** | 0.104 | 0.444 |
| **Age** | 0.010* | 0.090 | 0.020* | 0.000*** | 0.000*** | 0.003** | 0.000*** | 0.040* | 0.399 | 0.003** | 0.192 | 0.002** |
| **Education** | 0.203 | 0.747 | 0.165 | 0.604 | 0.816 | 0.136 | 0.825 | 0.297 | 0.195 | 0.274 | 0.417 | 0.281 |
| **Occupation** | 0.309 | 0.009** | 0.701 | 0.198 | 0.240 | 0.694 | 0.171 | 0.814 | 0.867 | 0.180 | 0.002** | 0.226 |
| **Household number** | 0.865 | 0.372 | 0.021* | 0.242 | 0.418 | 0.840 | 0.378 | 0.554 | 0.710 | 0.434 | 0.219 | 0.100 |
| **R^2^** | 0.018 | 0.018 | 0.032 | 0.033 | 0.055 | 0.034 | 0.029 | 0.008 | 0.016 | 0.047 | 0.025 | 0.028 |
| **Adjust R^2^** | 0.010 | 0.010 | 0.024 | 0.025 | 0.048 | 0.027 | 0.021 | 0.000 | 0.008 | 0.039 | 0.017 | 0.020 |
| **F statistic** | 2.209 | 2.208 | 4.041** | 4.241** | 7.274*** | 4.407** | 3.639** | 1.015 | 2.003 | 6.126*** | 3.172** | 3.541** |

****p<0.001, **p<0.01, *p<0.05*
