# Supplementary material for: Factors influencing the attention to home storage of medicines in China
Source: BMC Public Health. 2019 Jun 27;19:833. doi: 10.1186/s12889-019-7167-5 (PMC6598263; doi:10.1186/s12889-019-7167-5)
Supplement: Supplementary file 4 — Attention to home storage of medicines amongst different age groups. Table listing attention to home storage of medicines amongst different age groups. (DOCX 22 kb) [file 12889_2019_7167_MOESM4_ESM.docx]

**Additional file 1 Attention to home storage of medicines amongst different age groups**

|  |  | **18-30 years old**  **(n= 490 )** | | **31-45 years old**  **(n= 75 )** | | **46-60 years old**  **(n= 39)** | | **61-75 years old**  **(n= 17 )** | | **Above 75 years old**  **(n= 4 )** | |
| --- | --- | --- | --- | --- | --- | --- | --- | --- | --- | --- | --- |
| **Question** | **Attention levels** | **N** | **%** | **N** | **%** | **N** | **%** | **N** | **%** | **N** | **%** |
| **Q8** | Most attention | 167 | 34.1% | 33 | 44.0% | 21 | 53.8% | 11 | 64.7% | 2 | 50.0% |
|  | More attention | 150 | 30.6% | 18 | 24.0% | 5 | 12.8% | 4 | 23.5% | 1 | 25.0% |
|  | Attention | 109 | 22.2% | 15 | 20.0% | 8 | 20.5% | 1 | 5.9% | 0 | 0.0% |
|  | Some attention | 43 | 8.8% | 5 | 6.7% | 4 | 10.3% | 1 | 5.9% | 0 | 0.0% |
|  | No attention | 21 | 4.3% | 4 | 5.3% | 1 | 2.6% | 0 | 0.0% | 1 | 25.0% |
| **Q9** | Most attention | 170 | 34.7% | 34 | 45.3% | 18 | 46.2% | 12 | 70.6% | 2 | 50% |
|  | More attention | 152 | 31.0% | 16 | 21.3% | 9 | 23.1% | 3 | 17.6% | 0 | 0.0% |
|  | Attention | 101 | 20.6% | 13 | 17.3% | 6 | 15.4% | 1 | 5.9% | 1 | 25% |
|  | Some attention | 42 | 8.6% | 8 | 10.7% | 3 | 7.7% | 1 | 5.9% | 0 | 0.0% |
|  | No attention | 25 | 5.1% | 4 | 5.3% | 3 | 7.7% | 0 | 0.0% | 1 | 25.0% |
| **Q10** | Most attention | 33 | 6.7% | 6 | 8.0% | 4 | 10.3% | 6 | 35.3% | 0 | 0.0% |
|  | More attention | 67 | 13.7% | 18 | 24.0% | 11 | 28.2% | 4 | 23.5% | 0 | 0.0% |
|  | Attention | 138 | 28.2% | 19 | 25.3% | 6 | 15.4% | 1 | 5.9% | 2 | 50.0% |
|  | Some attention | 109 | 22.2% | 18 | 24.0% | 7 | 17.9% | 2 | 11.8% | 0 | 0.0% |
|  | No attention | 143 | 29.2% | 14 | 18.7% | 11 | 28.2% | 4 | 23.5% | 2 | 50.0% |
| **Q11** | Most attention | 77 | 15.7% | 25 | 33.3% | 9 | 23.1% | 9 | 52.9% | 0 | 0.0% |
|  | More attention | 107 | 21.8% | 26 | 34.7% | 13 | 33.3% | 3 | 17.6% | 1 | 25.0% |
|  | Attention | 138 | 28.2% | 10 | 13.3% | 6 | 15.4% | 2 | 11.8% | 2 | 50.0% |
|  | Some attention | 79 | 16.1% | 7 | 9.3% | 5 | 12.8% | 2 | 11.8% | 0 | 0.0% |
|  | No attention | 89 | 18.2% | 7 | 9.3% | 6 | 15.4% | 1 | 5.9% | 1 | 25.0% |
| **Q12** | Most attention | 28 | 5.7% | 7 | 9.3% | 5 | 12.8% | 9 | 52.9% | 0 | 0.0% |
|  | More attention | 41 | 8.4% | 16 | 21.3% | 5 | 12.8% | 1 | 5.9% | 0 | 0.0% |
|  | Attention | 134 | 27.3% | 14 | 18.7% | 12 | 30.8% | 0 | 0.0% | 2 | 50.0% |
|  | Some attention | 106 | 21.6% | 12 | 16.0% | 5 | 12.8% | 3 | 17.6% | 0 | 0.0% |
|  | No attention | 181 | 36.9% | 26 | 34.7% | 12 | 30.8% | 4 | 23.5% | 2 | 50.0% |
| **Q13** | Most attention | 64 | 13.1% | 13 | 17.3% | 8 | 20.5% | 10 | 58.8% | 0 | 0.0% |
|  | More attention | 100 | 20.4% | 17 | 22.7% | 13 | 33.3% | 2 | 11.8% | 1 | 25.0% |
|  | Attention | 156 | 31.8% | 18 | 24.0% | 8 | 20.5% | 2 | 11.8% | 1 | 25.0% |
|  | Some attention | 78 | 15.9% | 16 | 21.3% | 4 | 10.3% | 1 | 5.9% | 0 | 0.0% |
|  | No attention | 92 | 18.8% | 11 | 14.7% | 6 | 15.4% | 2 | 11.8% | 2 | 50.0% |
| **Q14** | Most attention | 131 | 26.7% | 30 | 40.0% | 17 | 43.6% | 12 | 70.6% | 2 | 50.0% |
|  | More attention | 130 | 26.5% | 21 | 28.0% | 13 | 33.3% | 3 | 17.6% | 0 | 0.0% |
|  | Attention | 137 | 28.0% | 14 | 18.7% | 5 | 12.8% | 1 | 5.9% | 0 | 0.0% |
|  | Some attention | 45 | 9.2% | 6 | 8.0% | 2 | 5.1% | 1 | 5.9% | 0 | 0.0% |
|  | No attention | 47 | 9.6% | 4 | 5.3% | 2 | 5.1% | 0 | 0.0% | 2 | 50.0% |
| **Q15** | Most attention | 198 | 40.4% | 33 | 44.0% | 24 | 61.5% | 11 | 64.7% | 0 | 0.0% |
|  | More attention | 142 | 29.0% | 21 | 28.0% | 10 | 25.6% | 3 | 17.6% | 2 | 50.0% |
|  | Attention | 87 | 17.8% | 16 | 21.3% | 3 | 7.7% | 0 | 0.0% | 1 | 25.0% |
|  | Some attention | 34 | 6.9% | 2 | 2.7% | 2 | 5.1% | 2 | 11.8% | 0 | 0.0% |
|  | No attention | 29 | 5.9% | 3 | 4.0% | 0 | 0.0% | 1 | 5.9% | 1 | 25.0% |
| **Q16** | Most attention | 83 | 16.9% | 18 | 24.0% | 11 | 28.2% | 8 | 47.1% | 0 | 0.0% |
|  | More attention | 103 | 21.0% | 20 | 26.7% | 7 | 17.9% | 4 | 23.5% | 1 | 25.0% |
|  | Attention | 157 | 32.0% | 14 | 18.7% | 5 | 12.8% | 1 | 5.9% | 0 | 0.0% |
|  | Some attention | 75 | 15.3% | 7 | 9.3% | 5 | 12.8% | 3 | 17.6% | 0 | 0.0% |
|  | No attention | 72 | 14.7% | 16 | 21.3% | 11 | 28.2% | 1 | 5.9% | 3 | 75.0% |
| **Q17** | Most attention | 65 | 13.3% | 13 | 17.3% | 9 | 23.1% | 9 | 52.9% | 1 | 25.0% |
|  | More attention | 63 | 12.9% | 12 | 16.0% | 7 | 17.9% | 2 | 11.8% | 0 | 0.0% |
|  | Attention | 133 | 27.1% | 20 | 26.7% | 7 | 17.9% | 1 | 5.9% | 1 | 25.0% |
|  | Some attention | 98 | 20.0% | 12 | 16.0% | 5 | 12.8% | 2 | 11.8% | 1 | 25.0% |
|  | No attention | 131 | 26.7% | 18 | 24.0% | 11 | 28.2% | 3 | 17.6% | 1 | 25.0% |
| **Q18** | Most attention | 51 | 10.4% | 13 | 17.3% | 6 | 15.4% | 7 | 41.2% | 0 | 0.0% |
|  | More attention | 119 | 24.3% | 15 | 20.0% | 9 | 23.1% | 4 | 23.5% | 1 | 25.0% |
|  | Attention | 181 | 36.9% | 32 | 42.7% | 13 | 33.3% | 2 | 11.8% | 0 | 0.0% |
|  | Some attention | 117 | 23.9% | 14 | 18.7% | 10 | 25.6% | 4 | 23.5% | 1 | 25.0% |
|  | No attention | 22 | 4.5% | 1 | 1.3% | 1 | 2.6% | 0 | 0.0% | 2 | 50.0% |
| **Q19** | Most attention | 125 | 25.5% | 23 | 30.7% | 15 | 38.5% | 2 | 11.8% | 0 | 0.0% |
|  | More attention | 101 | 20.6% | 12 | 16.0% | 3 | 7.7% | 3 | 17.6% | 1 | 25.0% |
|  | Attention | 141 | 28.8% | 14 | 18.7% | 7 | 17.9% | 1 | 5.9% | 0 | 0.0% |
|  | Some attention | 70 | 14.3% | 11 | 14.7% | 2 | 5.1% | 1 | 5.9% | 1 | 25.0% |
|  | No attention | 53 | 10.8% | 15 | 20.0% | 12 | 30.8% | 10 | 58.8% | 2 | 50.0% |
